# Ivermectin: A Closer Look at a Potential Remedy

**DOI:** 10.7759/cureus.10378

**Published:** 2020-09-11

**Authors:** Karim O Elkholy, Omar Hegazy, Burak Erdinc, Hesham Abowali

**Affiliations:** 1 Internal Medicine, Brookdale University Hospital Medical Center, Brooklyn, USA; 2 Internal Medicine, Mercy Hospital, Chicago, USA

**Keywords:** ivermectin, antiviral therapy, inhaled antivirals, remedisivir, favipiravir, hydroxychloroquine, severe acute respiratory syndrome coronavirus 2 (sars-cov-2), coronavirus 2019 (covid-19)

## Abstract

Amid the severe acute respiratory syndrome coronavirus 2 (SARS-CoV-2) pandemic, the search for effective treatment and vaccines has been exponentially on the rise. Finding effective treatment has been the core of attention of many scientific reports and antivirals are in the center of those treatments. Numerous antivirals are being studied for the management of the coronavirus disease 2019 (COVID-19) pneumonia caused by the SARS-CoV-2. Remdesivir was the first drug to gain emergency FDA approval to be used in COVID-19. Similarly, favipiravir, an anti-influenza drug, is being studied as a potential agent against COVID-19. Contrastingly, hydroxychloroquine has been a controversial drug in the management of COVID-19. Nevertheless, the National Institute of Health (NIH), along with the World Health Organization (WHO), have discontinued clinical trials for hydroxychloroquine as the drug showed little or no survival benefit. Ivermectin, an antihelminthic drug, has shown antiviral properties previously. Additionally, it was described to be effective in vivo against the SARS-CoV-2. However, its survival benefit in patients with COVID-19 has not been documented. We herein propose the theory of inhaled ivermectin which can attain the desired lung concentration that will render it effective against SARS-CoV-2.

## Introduction and background

**Figure 1:**
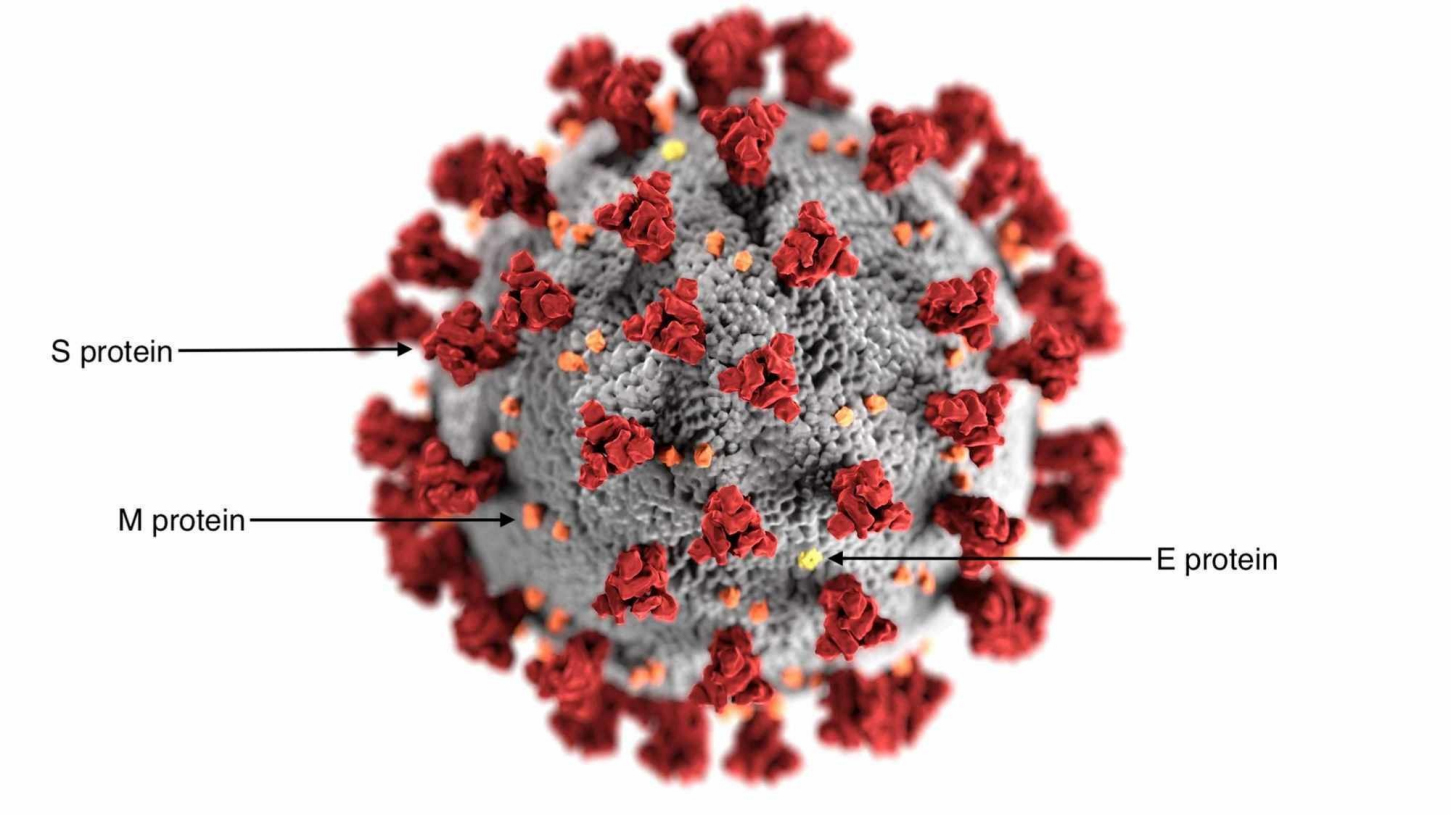
Severe acute respiratory syndrome coronavirus 2 (SARS-CoV-2) virus morphology This illustration, created at the Centers for Disease Control and Prevention (CDC), reveals ultrastructural morphology exhibited by coronaviruses. Note the spikes that adorn the outer surface of the virus, which impart the look of a corona surrounding the virion, when viewed electron microscopically. A novel coronavirus, named severe acute respiratory syndrome coronavirus 2 (SARS-CoV-2), was identified as the cause of an outbreak of respiratory illness first detected in Wuhan, China in 2019. The illness caused by this virus has been named coronavirus disease 2019 (COVID-19) [5].

The global pandemic caused by the severe acute respiratory syndrome coronavirus 2 (SARS-CoV-2) has affected more than 20 million people, claiming more than a half-million lives around the world [1]. As hopes for effective treatment and/or a vaccine rise every day, we are aiming here to contribute to the global goal of attaining effective treatment. Coronaviruses are enveloped, single-stranded, positive-sense ribonucleic acid (RNA) viruses that belong to the family *Coronaviridae* [2]. SARS-CoV-2 size usually ranges 70 - 90 nm, as shown in Figure 1, it consists of the surface viral protein spike, membrane, and envelope of coronavirus are embedded in the host membrane-derived lipid bilayer encapsulating the helical nucleocapsid comprising viral RNA [2]. The SARS-CoV-2 virus is the causative agent of the coronavirus disease 2019 (COVID-19) pneumonia. Looking at histopathological reports of eight patients, it was found that the SARS-CoV-2 virus caused diffuse alveolar damage (DAD) [3-4]. Overall, pathologic features in these eight COVID-19-related deaths were similar to those seen in severe acute respiratory syndrome coronavirus (SARS-CoV) and the Middle East respiratory syndrome coronavirus (MERS-CoV) infections [3-4]. However, SARS-CoV-2's extensive detection in epithelial cells of the upper respiratory tract is unique among these highly pathogenic coronaviruses [3-4].

## Review

A lot of potentially effective treatments were described in the literature for COVID-19 pneumonia. Antivirals have been the center of interest for many clinicians and scientists. 

Remdesivir 

Even though there are no drugs approved by the Food and Drug Administration (FDA) for the treatment of COVID-19, remdesivir, an investigational antiviral drug, is available through an FDA emergency use authorization. Remdesivir is a nucleotide analog inhibitor of RNA-dependent RNA polymerase (RdRps). More specifically, evidence of broad-spectrum antiviral activities against coronaviruses has been shown in vitro and animal models. In a recent study, this inhibitory action was demonstrated against the MERS-CoV where a delayed RNA chain termination was postulated as the likely mechanism of action [6]. The preliminary report described the efficiency of remdesivir where 1,063 patients underwent randomization for remdesivir (538) or placebo (521). The data showed patients receiving remdesivir had a median recovery time of 11 days as compared to 15 days in those who received a placebo (95% CI, 1.12 to 1.55; P < 0.001). It was also reported that Kaplan-Meir estimates of mortality by 14 days were lower in the remdesivir group (7.1%) compared to placebo (11.9%), However, these results were not statistically significant (95% CI, 0.47 to 1.04), rendering the mortality benefits of remdesivir still in question [7]. 

Hydroxychloroquine

Hydroxychloroquine, the anti-malarial medication, has been described in the management of SARS-CoV-2 infection [8-9]. It inhibits endocytic pathways through elevation of endosomal pH, altering enzyme production; however, the exact antiviral mechanism of action remains unclear. A significant benefit in the treatment of SARS-CoV-2 could not be established. Controversial data reports regarding this medication led to the halting of the National Institute of Health (NIH)-sponsored clinical trial for effects of hydroxychloroquine in the management of COVID-19 patients since the study showed treatment did no harm but also provided no benefit [10]. In addition to NIH, the World Health Organization (WHO) discontinued a clinical trial for hydroxychloroquine and lopinavir/ritonavir, as preliminary results concluded that those medications produced little or no reduction in the mortality of hospitalized COVID-19 patients when compared to the standard of care [11]. 

Favipiravir 

Favipiravir is a drug initially developed to treat severe cases of influenza in Japan. There are now ongoing clinical trials for its potential as an antiviral for SARS-CoV-2 [12]. Favipiravir directly inhibits viral replication and transcription via potent selective inhibition of the RNA-dependent RNA polymerase (RdRP) [13]. Cai et al. studied the effects of favipiravir in an open-label study involving 80 patients [14]. The study concluded that virus clearance was faster in the favipiravir arm (median: 4 days) compared to the control arm (median: 9 days) with P < 0.001. It also showed significant improvement in computerized tomography (CT) chest imaging when compared to control (91.43% vs 62.22%, P = 0.004) after adjustments for potential confounders. A shorter viral clearance time was found for the favipiravir arm (median: 4 days and interquartile range of 2.5 - 9 days) versus the control arm (median: 11 days with an interquartile range of 8 to 13 days). These results were statistically significant (P < 0.001) [14].

Ivermectin 

Ivermectin is an anti-helminthic drug that is used for the treatment of many parasitic infections which include head lice, scabies, river blindness (onchocerciasis), strongyloidiasis, and lymphatic filariasis. It can be administered orally or applied externally on the skin if needed [15]. It binds some channel proteins for chlorine (typical of specific classes of invertebrates), causing a greater permeability to this electrolyte and leading to blocking inhibitory neurotransmission in neurons and myocytes, resulting in paralysis and death [16]. Ivermectin has been described in the literature to have antiviral effects. Yang et al. identified that ivermectin molecule acts as an inhibitor of human immunodeficiency virus (HIV-1) integrase entry to the nucleus, consequently displaying that ivermectin could inhibit dengue virus (DENV) nonstructural protein 5 (NS5) nuclear entry as well, resulting in limiting infection by viruses, such as HIV-1 and DENV [17]. It would appear that ivermectin's broad-spectrum antiviral activity is related to its ability to target the host importin (IMP) α/β1, which are nuclear transport proteins responsible for HIV integrase and DENV NS5 entry to the nucleus [17]. The aforementioned report studied the effects of ivermectin on armadillo’s IMP α/β1 heterodimer. It was observed using quantitative bimolecular fluorescence complementation that ivermectin can dissociate the preformed IMPα/β1 heterodimer, in addition to preventing its formation through binding to the IMP α [17]. Severe acute respiratory syndrome coronavirus (SARS-CoV) is an enveloped single-stranded RNA virus. Timani et al. described the presence of N terminal nucleocapsid protein of SARS-CoV may act as a shuttle protein between cytoplasm and nucleolus [18]. Furthermore, Hiscox et al. described that the SARS-CoV nuclear protein (N) localizes both to the cytoplasmic and nucleolar compartments [19]. Porcine respiratory and reproductive syndrome virus (PRRSV, Arterivirus) is a virus similar to the *Coronaviridae* virus family in the context of being an enveloped RNA virus (Table 1). Wulan et al. described that disruption of nuclear/nucleolar localization of PRRSV nucleocapsid protein has been shown to attenuate viral replication and induce a higher titer of neutralizing antibodies in pigs [20]. The aforementioned reports suggest that nucleocytoplasmic transport inhibition by ivermectin might play an important role in its effectiveness against SARS-CoV-2.

**Table 1:** Related Enveloped RNA Viruses Classification

Virus Family	Subfamily	Genus
Flaviviridae	Flavivirus	Dengue fever, Japanese Encephalitis virus (JEV), West Nile virus (WNV)
	HepaCivirus	Hepatitis C
Coronaviridae	Alphacoronavirus	Transmissible gastroenteritis virus (TGEV)
	Betacoronavirus	Severe acute respiratory syndrome coronavirus (SARS-Cov), Mouse hepatitis virus
	Gammacoronavirus	Infectious bronchitis virus
Arteriviridae	Arterivirus	Lactate dehydrogenase elevating virus, equine arterivirus, porcine respiratory and reproductive syndrome virus (PRRSV)

Caly et al. described the efficiency of ivermectin against SARS-CoV-2 in vitro, where Vero/human signaling lymphocytic activation molecule (hSLAM) cells were infected with SARS-CoV-2 (Australia/VIC01/2020 isolate) at a multiplicity of infection (MOI) of 0.1 for two hours, followed by the addition of 5 μM ivermectin [21]. At 24 hours, there was a 93% reduction in the supernatant cells (indicative of released virions) and a 99.8% reduction in cell-associated viral RNA (indicative of unreleased and unpackaged virions) of samples treated with ivermectin compared to the control. By 48 hours, this effect increased to an ~5,000-fold reduction of viral RNA in ivermectin-treated samples compared to control. The half-maximal inhibitory concentration (IC50) of ivermectin treatment was determined to be ~2 μM under these conditions. These findings suggested a possible breakthrough in the management of SARS-CoV-2 infection. However, Schmith et al. proposed that the concentration resulting in IC50 reported being 2 uM was > 35x higher than the maximum plasma concentration (Cmax) after oral administration of the approved dose of ivermectin when given in a fasting state [22]. Predicted lung IC50, when given the approved dose of ivermectin, was 0.0857 uM. Nevertheless, at doses 10x higher than the approved dose, the predicted lung IC50 was 0.817 which remains below the IC50 for effective inhibition of viral replication. Ivermectin has demonstrated a safety profile in humans [15]. However, this report of ivermectin lung concentrations renders the achievement of effective IC50 in the lungs unlikely [22]. Moreover, Rajter et al. studied the effects of oral ivermectin in patients with COVID-19, the retrospective preprint report of 280 patients with confirmed COVID-19 infection found that there was lower mortality in the ivermectin group (15%) compared to the control group (25.2%) (95% CI 0.29 - 0.96, P = .03). After adjustment for the mortality risks between both groups, the mortality benefit remained significant for the study (hazard ratio (HR) 0.37, CI 0.19 - 0.71, p = .03) [23]. We hereby postulate the theory of achieving desired or close to the desired lung concentrations of ivermectin through a novel method of administration which is inhalation. 

Inhaled antivirals have been described in the literature. Billiard et al. described the efficiency and safety of inhaled ribavirin in healthy volunteers [24]. It was found that concentrations of ribavirin in the epithelial lining fluid (ELF) was achieved. ELF concentration of ribavirin was 101 µM and 112 µM when given a dose of 30 mg and 60 mg, respectively. Both concentrations correspond to a maximum plasma concentration (Cmax) of more than 300 µM. Biovin et al. studied the effects of inhaled zanamivir in the treatment of influenza in otherwise healthy individuals [25]. It was observed that there was a 1.0 log_10_ TCID_50_/mL decrease in median viral titers compared to 0.42 log_10_ increase in the placebo group (p = 0.08) [25]. Those reports can predict a possible and attainable IC_50_ lung concentration of ivermectin if it is given through inhalation, which indicates that inhaled ivermectin should be evaluated as a potential broad-spectrum antiviral against respiratory viruses. 

Inhaled medications are affected by several factors. As shown in Table 2, Borghardt et al. summarized those factors into (1) drug particle or droplet deposition, (2) drug dissolution in the lung fluids, (3) mucociliary clearance in the conducting airways and macrophage clearance in the alveolar space, (4) absorption to lung tissue, (5) pulmonary tissue retention and potential pulmonary metabolism, and (6) absorptive drug clearance (drug transport) from the lung tissue to the systemic perfusion [26].

**Table 2:** Factors Affecting Drugs for Inhalation

Factors	Generally	Ivermectin
Drug particle or droplet deposition	Aerodynamic diameter of approximately 0.5 – 5 µm have the greatest potential to be deposited in the lung	Manufacturer-controlled factor
Drug dissolution in the lung fluids	The conducting airways are lined with a biphasic gel-aqueous mucus layer that may act as a barrier. Alveoli are lined with alveolar lining fluid and pulmonary surfactant can facilitate dissolution	Highly lipid-soluble with presumed prolonged lung retention [27]
Mucociliary clearance in the conducting airways and macrophage clearance in the alveolar space	The velocity of mucociliary clearance increases with a wider airway diameter and a thicker mucus layer. It is fastest in the large airways Therefore, drug particles initially deposited in the central airways are cleared most quickly.	Patient and drug-dependent factor.
Absorption to lung tissue	Lipophilic drugs are rapidly absorbed by passive transcellular diffusion through epithelial cells. Hydrophilic drugs are absorbed by paracellular diffusion across the epithelium may occur through aqueous pores in intercellular gap junctions	Lipid-soluble drug with presumed rapid absorption after dissolution [27]
Pulmonary tissue retention and potential pulmonary metabolism	Basic drug molecules are reported to be retained in the lung by lysosomal trapping	Patient and drug-dependent factors
Absorptive drug clearance (drug transport) from the lung tissue to the systemic perfusion	Levels of local perfusion vary between the different structures in the lung and are highest in the alveolar region.	Patient-dependent factor.

During our literature review, we found one report studying the effect of inhaled ivermectin on rats, exposing rats to four hours of inhaled ivermectin five days per week for four weeks. It was noticed that the no observed adverse effect level (NOAEL) was determined at 380 mg/m^3^ [28].

## Conclusions

Based on the above-mentioned reports, we see that the development of an inhaled form of ivermectin needs further attention, given its potential efficacy in the treatment of COVID-19. The use of inhaled ivermectin as a potential antiviral medication for the SARS-CoV-2 virus might be an easy and affordable solution while facing a global pandemic. Ivermectin could have broad-spectrum antiviral properties which can lead to its potential use for other respiratory viral infections, rendering it effective in managing emerging viral outbreaks in the future. The need for further studies to investigate the efficiency and safety of inhaled ivermectin in the human population is warranted.
